# Imino chemical shift assignments of tRNA^Asp^, tRNA^Val^ and tRNA^Phe^ from *Escherichia coli*

**DOI:** 10.1007/s12104-024-10207-0

**Published:** 2024-10-04

**Authors:** Marcel-Joseph Yared, Carine Chagneau, Pierre Barraud

**Affiliations:** Expression génétique microbienne, Université Paris Cité, CNRS, Institut de biologie physico-chimique, IBPC, 13 rue Pierre et Marie Curie, Paris, 75005 France

**Keywords:** tRNA, Imino groups, Amino groups, NMR, RNA chemical shift assignments, Post-transcriptional modifications

## Abstract

Transfer RNAs (tRNAs) are an essential component of the protein synthesis machinery. In order to accomplish their cellular functions, tRNAs go through a highly controlled biogenesis process leading to the production of correctly folded tRNAs. tRNAs in solution adopt the characteristic L-shape form, a stable tertiary conformation imperative for the cellular stability of tRNAs, their thermotolerance, their interaction with protein and RNA complexes and their activity in the translation process. The introduction of post-transcriptional modifications by modification enzymes, the global conformation of tRNAs, and their cellular stability are highly interconnected. We aim to further investigate this existing link by monitoring the maturation of bacterial tRNAs in *E. coli* extracts using NMR. Here, we report on the ^1^H, ^15^N chemical shift assignment of the imino groups and some amino groups of unmodified and modified *E. coli* tRNA^Asp^, tRNA^Val^ and tRNA^Phe^, which are essential for characterizing their maturation process using NMR spectroscopy.

## Biological context

Transfer RNAs (tRNAs) are small non-coding RNAs that are conserved, in terms of structure and function, in all domains of life. They exhibit a distinctive cloverleaf secondary configuration comprising the T-arm, D-arm, anticodon arm, variable region and acceptor stem. Tertiary interactions between the T- and D-loops, thereby forming the tRNA elbow, and between the variable region and the D-stem lead to the characteristic L-shaped three-dimensional structure of tRNAs (Robertus et al. 1974; Suddath et al. 1974). The correct assembly and folding of these structural components are vital for the tRNA to properly perform all of its versatile functions in translation (Agris et al. 2017; Smith et al. 2024) and in other cellular processes (Raina and Ibba 2014; Fields and Roy 2018). In order to respond to this evolutionary pressure, tRNAs go through a tightly controlled biogenesis process leading to the formation of properly folded, mature and functional tRNAs. One particular maturation step is the introduction of chemical modifications on specific tRNA nucleotides making tRNAs the most extensively and diversely modified RNAs in the cell (Cappannini et al. 2024). Modifications introduced around the anticodon loop are mainly implicated in translation fidelity and efficiency (El Yacoubi et al. 2012; Agris et al. 2017; Smith et al. 2024), while modifications introduced in the core region are mainly implicated in the folding and stability of tRNAs (Motorin and Helm 2010; Lorenz et al. 2017; Yared et al. 2024). Since all aspects of tRNA biology are affected by modifications to some extent, the introduction of these modifications is tightly regulated and controlled by multiple factors (Barraud and Tisné 2019). Indeed, several modification circuits have been identified in which one or more modifications stimulate or repress the incorporation of subsequent modifications (Sokołowski et al. 2018; Han and Phizicky 2018; Barraud and Tisné 2019; Yared et al. 2024). In the past years, we developed an NMR-based methodology to monitor the maturation of tRNAs in a time resolved fashion (Gato et al. 2021). This led to the identification of several modification circuits present in the core of yeast tRNA^Phe^ (Barraud et al. 2019). We now aim at expanding this methodology to study the maturation of bacterial tRNAs, in order to identify potential modifications circuits associated with their maturation process.

**Fig. 1:**
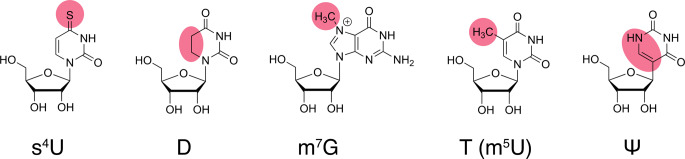
Chemical structure of common modified residues discussed in the assignment procedures. 4-thiouridine (s^4^U), dihydrouridine (D), 7-methylguanosine (m^7^G), thymidine or 5-methyluridine (T or m^5^U), and pseudouridine (Ψ). Modifications are highlighted in red

Here, we describe the production and purification of unmodified and modified samples of *E. coli* tRNA^Asp(GUC)^, tRNA^Val(UAC)^ and tRNA^Phe(GAA)^, and we report on the ^1^H, ^15^N chemical shift assignment of their imino groups and of some amino groups of tRNA^Asp(GUC)^. Unmodified samples correspond to in vitro transcribed tRNAs that do not carry any post-transcriptional modifications, whereas modified samples correspond to fully modified tRNAs overexpressed and purified from *E. coli* (see Fig. 1 for the chemical structure of the modifications present in these tRNAs and mentioned in the assignment procedures). The resonance assignments and the comparison of the imino groups chemical shifts of the unmodified and modified sample of each tRNA, revealed the NMR signature of individual post-transcriptional modifications needed for the subsequent NMR study of the maturation of these tRNAs in a time-resolved fashion.

## Methods

### Sample preparation

Unmodified *E. coli* tRNA^Asp(GUC)^, tRNA^Val(UAC)^, and tRNA^Phe(GAA)^ were prepared by standard in vitro transcription either with unlabeled NTPs (Jena Bioscience) or ^15^N-labeled GTP and UTP (Cambridge Isotope Laboratories) (Table 1). Transcription mix contains Tris-HCl pH 8.0 20 mM, spermidine 0.5 mM, DTT 2.5 mM, Triton X-100 at 0.005% (v/v), 200 nM of an equimolar mixture of the DNA template and T7 promotor primer, each of the 4 NTPs at 5 mM, MgCl_2_ 40 mM, GMP 8 mM and 25 µg/mL of in-house produced T7 RNA polymerase containing a mutation (Pro266Leu) for improved efficiency (Guillerez et al. 2005). tRNA^Asp^, tRNA^Val^, and tRNA^Phe^ were transcribed and purified following a previously published procedure (Catala et al. 2020a, b; Yared et al. 2023). Briefly, all unmodified tRNA samples were purified by anion exchange chromatography while unmodified tRNA^Asp^ and tRNA^Val^ were further purified using a hydrophobic interaction column (Phenyl Superose HR 10/10 column) equilibrated in 25 mM Na-phosphate pH 6.5 and 1.7 M ammonium sulfate. They were then eluted using a 1.7-0 M ammonium sulfate reverse gradient. tRNAs were then extensively dialysed against 1 mM of Na-phosphate pH 6.0 buffer and refolded at 95 °C for 5 min. After slowly cooling down the samples, buffer was added to place each tRNA in the NMR buffer (Na-phosphate pH 6.0 10 mM, MgCl_2_ 10 mM). Finally, tRNA samples were concentrated to a ∼ 0.2-1 mM using Amicon 10,000 MWCO (Millipore).

**Table 1:** The NMR experiments used in the assignment of tRNA^Asp^, tRNA^Val^, tRNA^Phe^ and the corresponding acquisition parameters. Unlabeled samples correspond to unmodified and modified tRNAs prepared using unlabeled NTPs or media. ^15^N-UG samples correspond to in vitro transcribed tRNAs using ^15^N-labeled GTP and UTP. u-^15^N samples correspond to uniformly ^15^N-labeled modified tRNAs produced in ^15^N-labeled media

tRNA sample		Experiment	Temp	Field (MHz)	τ_m_ (ms)	D1 (s)	NS	Points (F2, F1)	SW (ppm)	AQ (ms)	Carrier frequencies (ppm)
unmodified tRNA^Asp^	unlabeled 0.55 mM	(^1^H,^1^H)-NOESY	311 K	700	150	1.0	256	4096, 744	27.9 (^1^H), 21.6 (^1^H)	105 (^1^H), 24.6 (^1^H)	4.7 (^1^H), 4.7 (^1^H)
	^15^N-UG 0.27 mM	(^1^H,^15^N)-BEST-TROSY	311 K	700	-	0.2	32	4096, 128	25.9 (^1^H), 26 (^15^N)	113 (^1^H), 34.7 (^15^N)	4.7 (^1^H), 154.5 (^15^N)
modified tRNA^Asp^	u-^15^N 0.43 mM	(^1^H,^1^H)-NOESY	311 K	700	150	1.0	296	4096, 744	27.9 (^1^H), 21.6 (^1^H)	105 (^1^H), 24.6 (^1^H)	4.7 (^1^H), 4.7 (^1^H)
		(^1^H,^15^N)-BEST-TROSY	311 K	700	-	0.2	196	4096, 256	25.9 (^1^H), 26 (^15^N)	113 (^1^H), 69.4 (^15^N)	4.7 (^1^H), 154.5 (^15^N)
		(^1^H,^15^N)-BEST-TROSY-HNN-COSY	311 K	700	-	0.3	512	3072, 324	25.9 (^1^H), 118 (^15^N)	84.8 (^1^H), 19.3 (^15^N)	4.7 (^1^H), 185 (^15^N)
		(^1^H,^15^N)-HSQC-CPMG-NOESY	311 K	700	130	1.1	200	2714, 404	25.9 (^1^H), 110 (^15^N)	74.9 (^1^H), 25.8 (^15^N)	4.7 (^1^H), 116 (^15^N)
		(^1^H,^15^N)-HSQC	311 K	700	-	1.0	180	2048, 280	18 (^1^H), 40 (^15^N)	81.1 (^1^H), 49.3 (^15^N)	4.7 (^1^H), 87 (^15^N)
unmodified tRNA^Phe^	unlabeled 1.0 mM	(^1^H,^1^H)-NOESY	311 K	950	200	1.0	128	5120, 760	23.9 (^1^H), 20 (^1^H)	113 (^1^H), 20 (^1^H)	4.7 (^1^H), 4.7 (^1^H)
	^15^N-UG 0.45 mM	(^1^H,^15^N)-BEST-TROSY	311 K	950	-	0.2	36	4096, 220	28.2 (^1^H), 26 (^15^N)	76.4 (^1^H), 43.9 (^15^N)	4.7 (^1^H), 154.5 (^15^N)
modified tRNA^Phe^	unlabeled 0.53 mM	(^1^H,^1^H)-NOESY	311 K	950	200	1.0	128	5120, 760	23.9 (^1^H), 20 (^1^H)	113 (^1^H), 20 (^1^H)	4.7 (^1^H), 4.7 (^1^H)
	u-^15^N 0.02 mM	(^1^H,^15^N)-BEST-TROSY	311 K	950	-	0.2	212	4096, 144	28.2 (^1^H), 26 (^15^N)	76.4 (^1^H), 28.8 (^15^N)	4.7 (^1^H), 154.5 (^15^N)
		(^1^H,^15^N)-HSQC	311 K	700	-	1.0	128	3072, 168	25 (^1^H), 26 (^15^N)	87.8 (^1^H), 45.5 (^15^N)	4.7 (^1^H), 154.5 (^15^N)
unmodified tRNA^Val^	unlabeled 0.32 mM	(^1^H,^1^H)-NOESY	298 K	700	150	1.0	256	4096, 744	27.9 (^1^H), 21.6 (^1^H)	105 (^1^H), 24.6 (^1^H)	4.7 (^1^H), 4.7 (^1^H)
	^15^N-UG 0.2 mM	(^1^H,^15^N)-BEST-TROSY	298 K	700	-	0.2	40	4096, 128	25.9 (^1^H), 26 (^15^N)	113 (^1^H), 34.7 (^15^N)	4.7 (^1^H), 154.5 (^15^N)
modified tRNA^Val^	unlabeled 1.25 mM	(^1^H,^1^H)-NOESY	298 K	700	150	1.0	120	4096, 568	27.9 (^1^H), 21.6 (^1^H)	105 (^1^H), 18.8 (^1^H)	4.7 (^1^H), 4.7 (^1^H)
		(^1^H,^15^N)-BEST-TROSY	298 K	700	-	0.2	9000	4096, 72	25.9 (^1^H), 26 (^15^N)	113 (^1^H), 19.5 (^15^N)	4.7 (^1^H), 154.5 (^15^N)

The modified samples of *E. coli* tRNA^Asp(GUC)^, tRNA^Val(UAC)^, and tRNA^Phe(GAA)^ were produced in and purified from *E. coli* JM101TR strain following previously published procedures (Catala et al. 2020a, b). The genes coding for the tRNA^Asp^, tRNA^Val^ and tRNA^Phe^ were cloned each in the pBSTNAV vector between the *EcoRI* and *PstI* restriction sites and then expressed in 2xTY growth medium for unlabeled samples or in ^15^N-labeled Spectra-9 medium (Eurisotop) for ^15^N-labeled samples. After standard procedures of phenol extraction, each tRNA was first purified by size exclusion chromatography on a Superdex 75 increase 10/300 GL equilibrated in 25 mM K-phosphate pH 6.5. Then the eluted samples were purified by anion exchange chromatography (MonoQ, GE Healthcare) equilibrated in 25 mM K-phosphate pH 6.5 and eluted using a 450–600 mM NaCl gradient in the same buffer. The modified samples of tRNA^Phe^ were further purified by hydrophobic interaction chromatography using Phenyl superose HR 10/10 equilibrated in 25 mM K-phosphate pH 6.5 and 1.7 M ammonium sulfate. tRNA^Phe^ was then eluted using a 1.4–0.9 M ammonium sulfate reverse gradient. After each purification step, fractions containing the overexpressed tRNA were identified with a gel-shift assay on a native PAGE with a DNA oligonucleotide complementary to a portion of the T-arm, the anticodon stem-loop and a portion of the D-arm as previously described (Catala et al. 2020b). All samples were then dialyzed against the NMR buffer and concentrated using Amicon 10,000 MWCO (Millipore).

### NMR spectroscopy

NMR spectra of *E. coli* tRNA^Asp^ and tRNA^Val^ were measured at 311 K and 298 K, respectively, on Bruker AVIII-HD 700 MHz spectrometer and NMR spectra of *E. coli* tRNA^Phe^ were measured at 311 K on Bruker AVIII-HD 700 and 950 MHz spectrometers, all with 5-mm Shigemi tubes (Table 1). Imino resonances of tRNA^Asp^, tRNA^Val^ and tRNA^Phe^ were assigned using 2D jump-and-return-echo (^1^H,^1^H)-NOESY (Plateau and Gueron 1982; Sklenar and Bax 1987) and 2D (^1^H,^15^N)-BEST-TROSY (Farjon et al. 2009) (Table 1). Amino resonances of modified tRNA^Asp^ were assigned using (^1^H,^15^N)-HSQC-CPMG-NOESY (Mueller et al. 1995) and standard (^1^H,^15^N)-HSQC experiments (Table 1). Lastly, detection of hydrogen bonds in base pairs was performed using a (^1^H,^15^N)-BEST-TROSY-HNN-COSY experiment (Dallmann and Sattler 2014; Dallmann et al. 2013), thereby providing information on the type of base-pairing. All experiments were measured in the NMR buffer supplemented with 5% (v/v) of D_2_O. The sample concentrations and the NMR acquisition parameters are listed in Table 1. The data were processed using TOPSPIN 3.6 (Bruker) and analysed with NMRFAM-SPARKY (Lee et al. 2015). The spectra were referenced to 2,2-dimethyl-2-silapentanesulfonic acid (DSS) using an external sample of 0.5% DSS and 2 mM sucrose in H_2_O/D_2_O (Bruker), and indirect chemical shift referencing for ^15^N according to IUPAB (Markley et al. 1998).

### Extent of assignment and data deposition

**Fig. 2:**
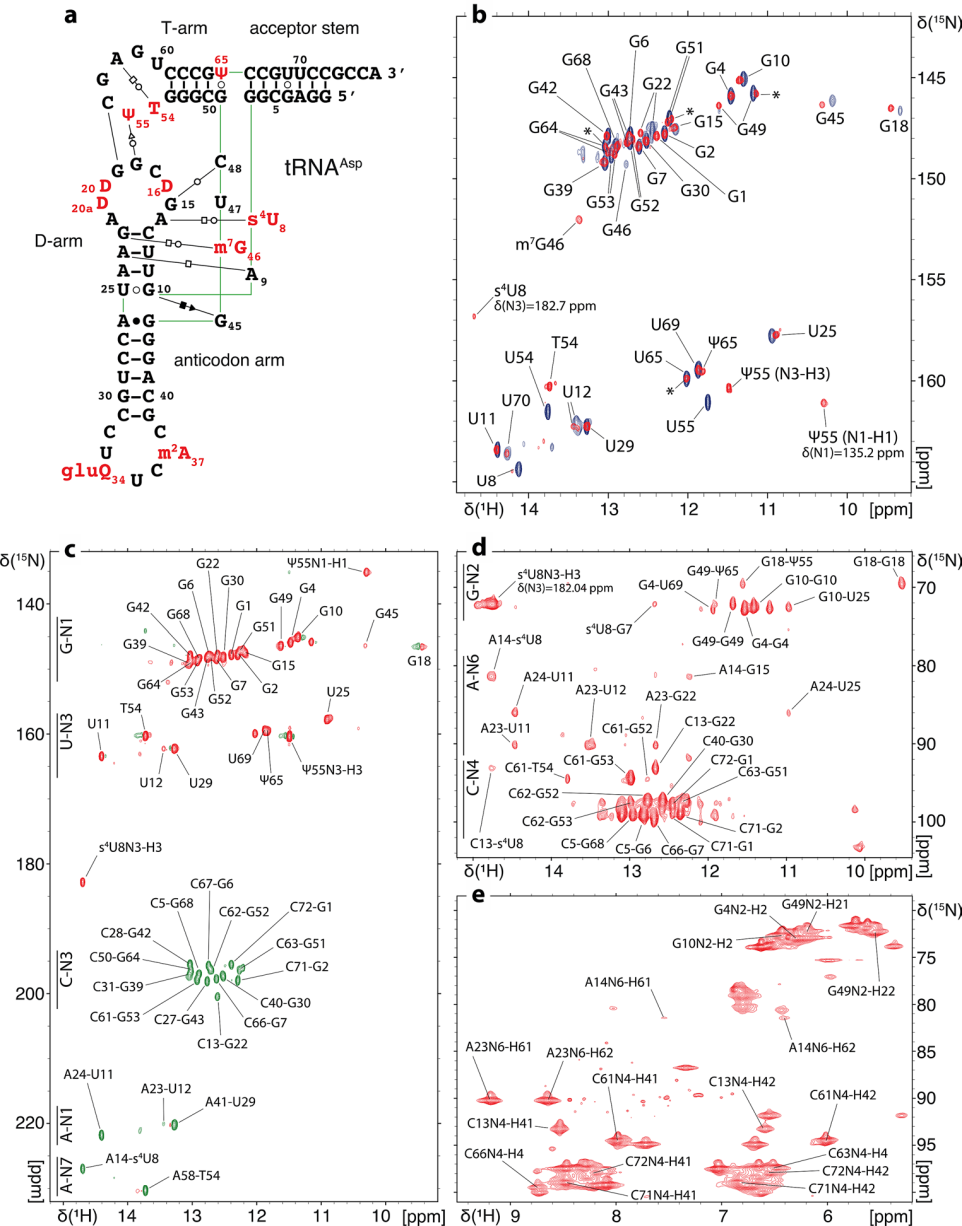
^1^H, ^15^N chemical shift assignment of the imino and amino groups of *E. coli *tRNA^Asp(GUC)^. (**a**) Sequence and L-shape 2D representation of modified *E. coli* tRNA^Asp(GUC)^. Modified residues are represented in red. The nature of interactions between base pairs is described according to the classification by Leontis and Westhof (Leontis and Westhof 2001). (**b**) (^1^H,^15^N)-BEST-TROSY spectrum and assignment of the imino groups of unmodified and modified *E. coli* tRNA^Asp^. Signals from the unmodified sample are represented in blue and signals from the modified sample are in red. Asterisks denote peaks in the modified sample that correspond to a subpopulation in which U65 is not modified into Ψ65. (**c**) (^1^H,^15^N)-BEST-TROSY-HNN-COSY spectrum showing correlations across hydrogen bonds in the modified tRNA^Asp^. (**d**) (^1^H,^15^N)-CPMG-NOESY-HSQC enabling the chemical shift assignment of the amino groups in the modified tRNA^Asp^. (**e**) (^1^H,^15^N)-HSQC spectrum showing the chemical shift assignment of the NH_2_-amino groups of modified tRNA^Asp^

**Fig. 3:**
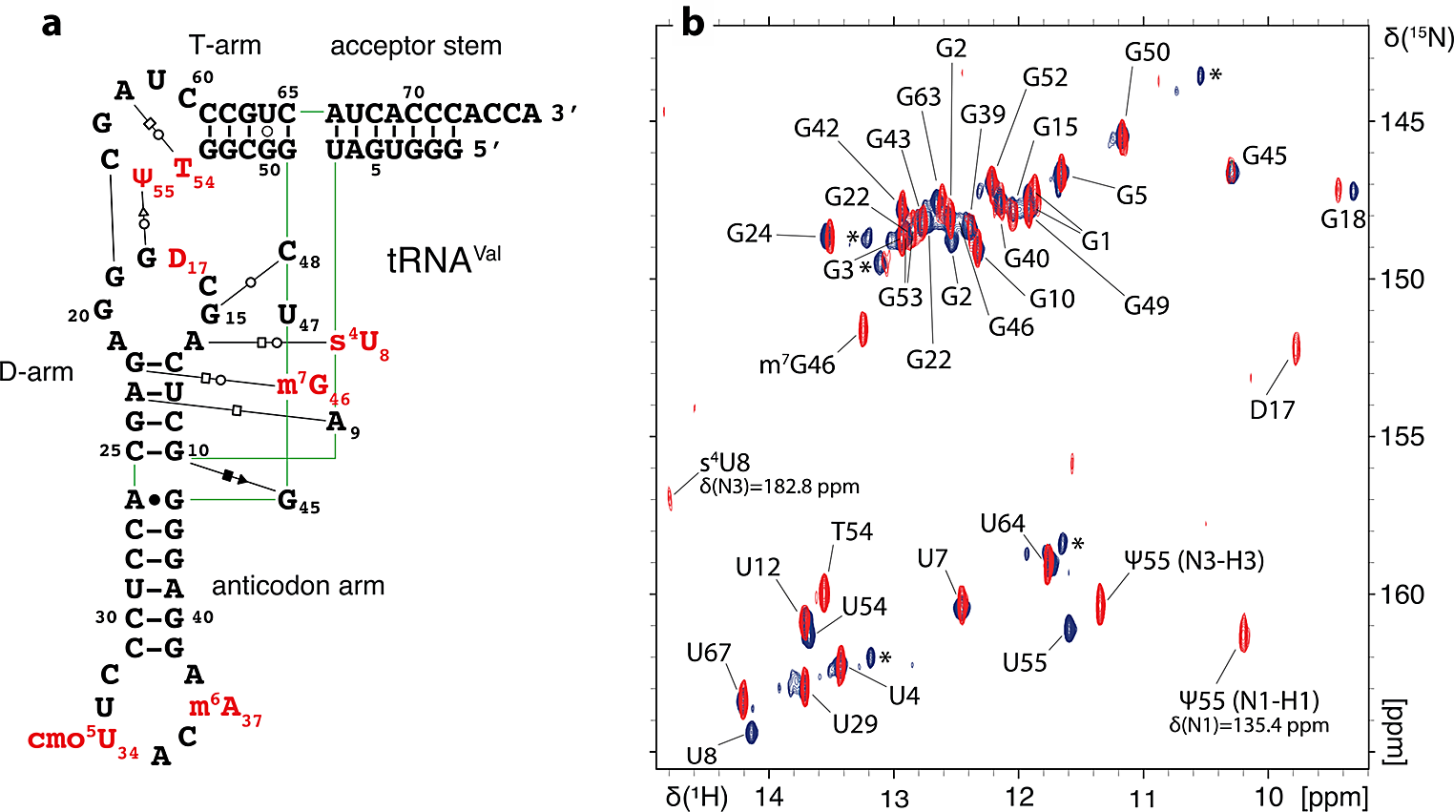
^1^H,^15^N chemical shift assignment of the imino groups of *E. coli *tRNA^Val(UAC)^. (**a**) Sequence and L-shape 2D representation of modified *E. coli* tRNA^Val(UAC)^. The same code is used as on Fig. 2. (**b**) (^1^H,^15^N)-BEST-TROSY spectrum and assignment of the imino groups of unmodified and modified *E. coli* tRNA^Val^. Signals from the unmodified sample are represented in blue and signals from the modified sample are represented in red. Asterisks denote peaks that correspond to an alternative folding of unmodified tRNA^Val^

**Fig. 4:**
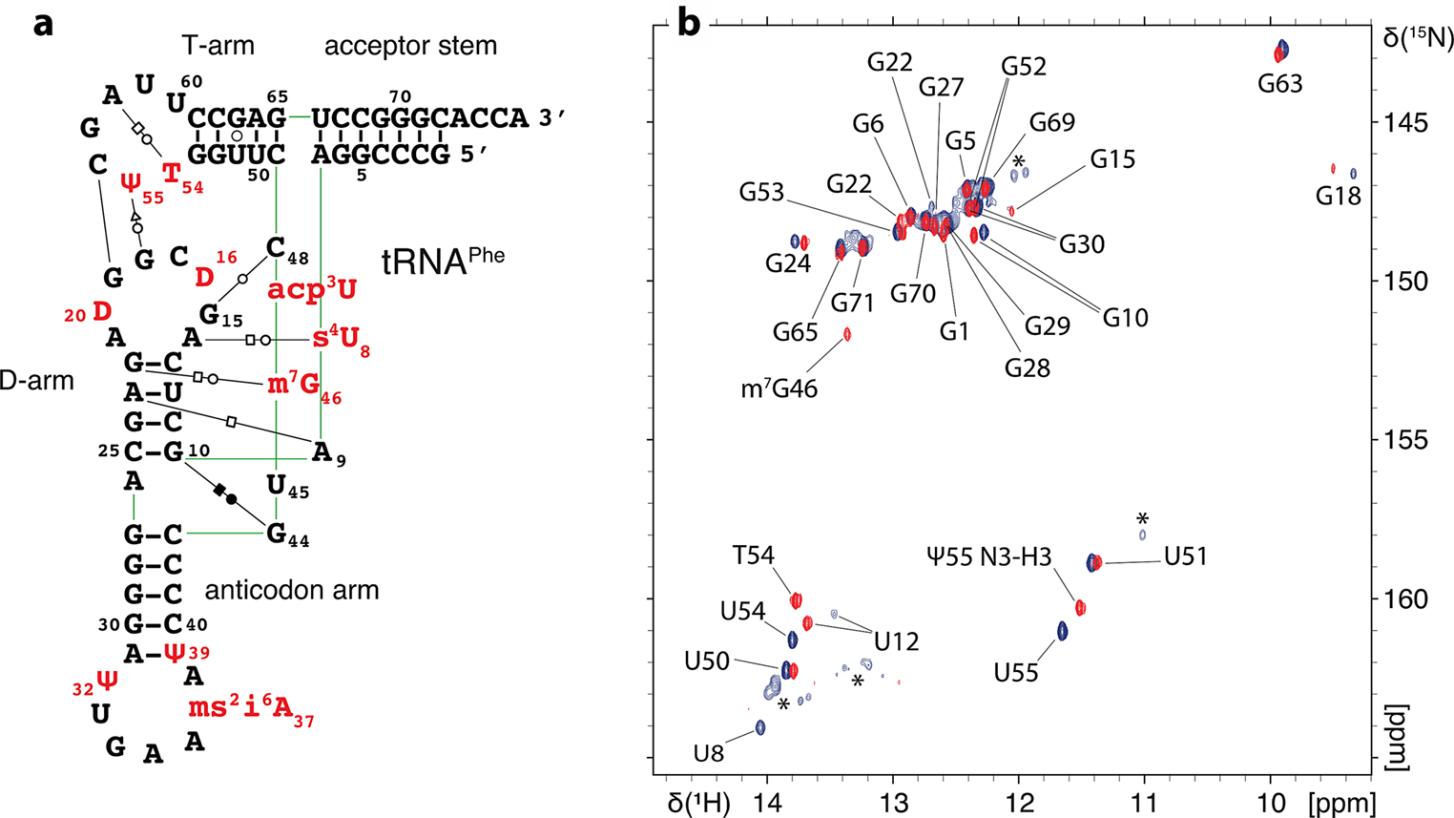
^1^H,^15^N chemical shift assignment of the imino groups of *E. coli *tRNA^Phe(GAA)^. (**a**) Sequence and L-shape 2D representation of modified *E. coli* tRNA^Phe(GAA)^. The same code is used as on Fig. 2. (**b**) (^1^H,^15^N)-BEST-TROSY spectrum and assignment of the imino groups of unmodified and modified *E. coli* tRNA^Phe^. Signals from the unmodified sample are represented in blue and signals from the modified sample are represented in red. Asterisks denote minor peaks in the unmodified tRNA^Phe^ corresponding to minor alternative folding conformations

**Fig. 5:**
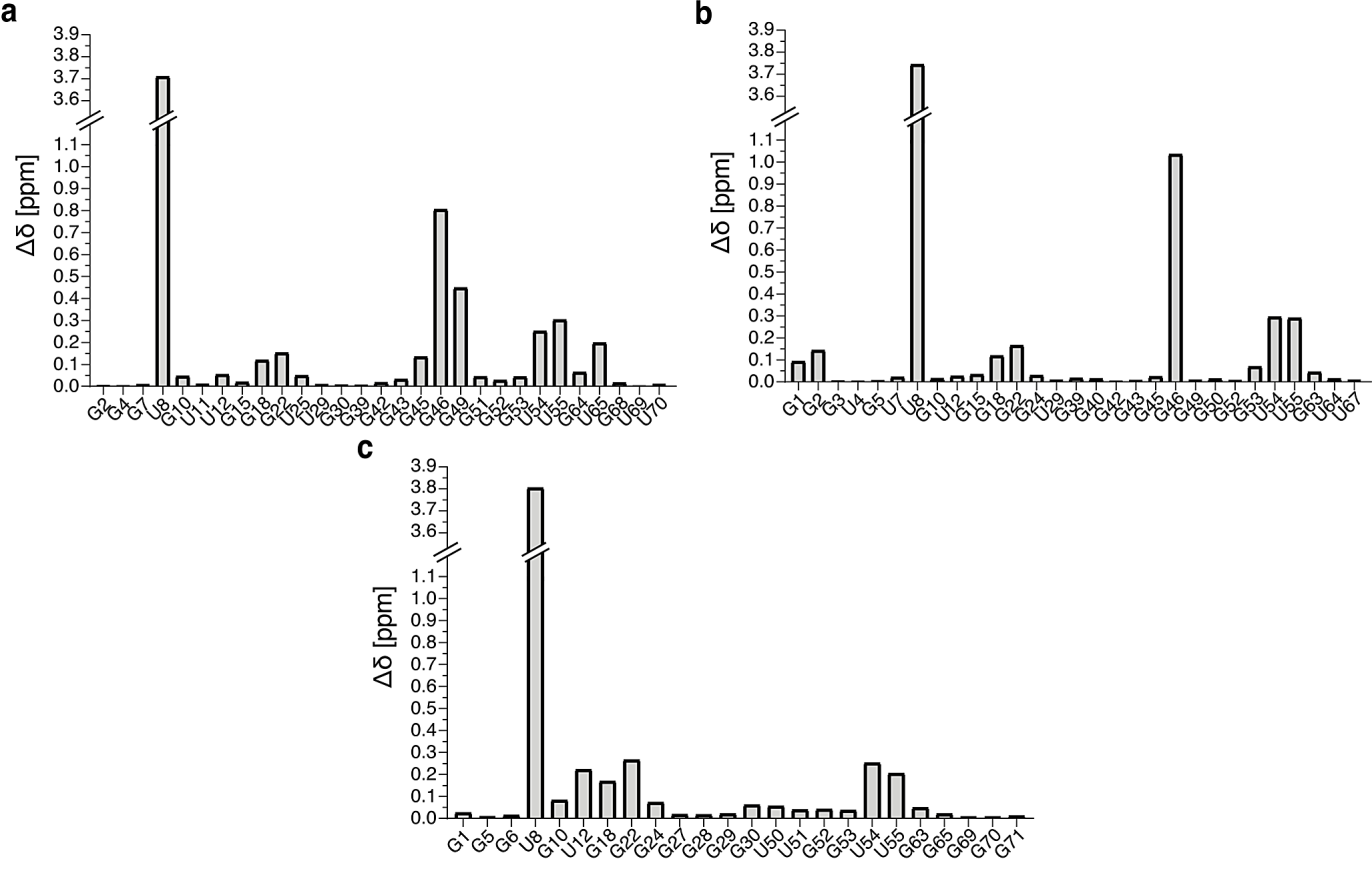
^1^H and ^15^N combined chemical shift differences between the imino resonances of the unmodified and modified *E. coli* tRNAs. Histograms showing the combined chemical shifts difference of assigned nucleotides between unmodified and modified samples of (**a**) tRNA^Asp(GUC)^, (**b**) tRNA^Val(UAC)^ and (**c**) tRNA^Phe(GAA)^

In order to select the most appropriate temperature for the detection of imino proton signals, we initially measured 1D jump-and-return-echo NMR spectra at different temperatures ranging from 293 K to 311 K on each of the unmodified tRNA samples. The temperature of NMR measurement was subsequently fixed at 311 K for tRNA^Asp^ and tRNA^Phe^ and at 298 K for tRNA^Val^. 2D (^1^H,^1^H)-NOESY and 2D (^1^H,^15^N)-BEST-TROSY spectra have been obtained from unmodified and modified samples, which led to the imino groups resonance assignments of the unmodified and modified guanines and uridines that are protected from the exchange with the solvent (Figs. 2, 3 and 4). In addition, methyl groups of T54 (m^5^U54) and m^7^G46 were assigned and used as starting points or as means of verifying the assignments consistency. (^1^H,^15^N)-HSQC-CPMG-NOESY and standard (^1^H,^15^N)-HSQC experiments were used to assign the amino resonances of modified tRNA^Asp^. As a general principle, we performed the assignment of the unmodified and modified versions of each tRNA together to support the assignments with internal consistencies. Unless otherwise stated, the chemical shifts of imino groups reported in the text are from TROSY-type experiments and correspond to Figs. 2b, 3b and 4b, while the chemical shifts deposited in the BioMagResBank correspond to isotropic chemical shifts. Overall, we have followed a similar strategy for assigning the different tRNAs, starting from the assignment of the D-arm and variable region, followed by the assignment of the T-arm and acceptor stem, and finally by the anticodon stem. The assignment processes are described below following this order, with dedicated paragraphs for each tRNA. In addition, the differences in the combined ^1^H and ^15^N chemical shifts of assigned nucleotide between the unmodified and modified samples of tRNA^Asp^, tRNA^Val^ and tRNA^Phe^ were determined using the following equation:$\:\varDelta\:\delta\:=\sqrt{{\left(\varDelta\:{\delta\:}^{1}\text{H}\right)}^{2}+{\left(\varDelta\:{\delta\:}^{15}\text{N}/5\right)}^{2}}$ (Fig. 5).

### Assignment of *E. coli* tRNA^Asp^

For the assignment of tRNA^Asp^, we relied on the 3D structure of the unmodified tRNA to identify tertiary interactions and to determine the spatial proximity between detectable nuclei (Chan et al. 2020) (Fig. 2a).

In order to assign the imino resonances stemming from the D-arm (Fig. 2b), we took U8 and s^4^U8 as starting points. Typically, the imino resonance of U8 is the most downfield shifted peak. In addition, s^4^U8 in modified samples exhibit a downfield shifted ^15^N frequency characteristic of the s^4^U modification at around 183 ppm. Based on the observed intra-base pair NOEs, s^4^U8 connects to iminos of m^7^G46, G15 and G22. Moving forward from G22, we managed to assign the remaining iminos in the D-arm (Fig. 2b). In addition, the imino proton of m^7^G46 connects with iminos of G22, G15 and U12 since m^7^G46 is involved in a tertiary interaction with G22 connecting the variable loop with the D-arm (Fig. 2a). Imino groups of U25, G10 and U11 in the D-arm connect weakly with the imino of a guanine, the latter lacking NOEs with other residues in its close environment. Based on tRNA^Asp^ tertiary structure, nucleotides G44 and G45 are in the near environment of U25, G10 and U11 with G45 being the closest (Chan et al. 2020) (Fig. 2a). G45 forms with U25 and G10 the base triplet G10-U25-G45. Each amino proton of G45 is involved in a hydrogen bond with O6 and N7 of G10 respectively. G44 forms a non-canonical base pair with A26 with its imino proton involved in a hydrogen bond with N1 of A26. In order to assign this guanine and distinguish between G44 and G45, we measured a (^1^H,^15^N)-BEST-TROSY-HNN-COSY experiment on a uniformly ^15^N-labeled modified tRNA^Asp^ overexpressed in *E. coli* (Fig. 2c). The imino group of G45 should not correlate with any nitrogen since it is not involved in a hydrogen bond with a nitrogen atom, while we expect the N1 of G44 to correlate with the N1 of A26. We do not observe any detectable correlation with this guanine in the (^1^H,^15^N)-BEST-TROSY-HNN-COSY (Fig. 2c). In addition, a peak with similar chemical shifts was assigned to G45 in *E. coli* tRNA^Ile^ (Jesus et al. 2022). Altogether, we opt for assigning this guanine as G45.

In addition, with the HNN-COSY experiment, we managed to correlate the N1 of guanines to the N3 of cytosines and the N3 of uridines to N1 and N7 of adenosines (Fig. 2c). This serves as a cross-validation of the assignment of certain nucleotides involved in non-Watson-Crick interactions (Fig. 2a, c).

With the modified and uniformly ^15^N-labeled tRNA^Asp^ sample, we could in addition assign some amino groups of paired Gs, As, and Cs of tRNA^Asp^ by measuring a (^1^H,^15^N)-HSQC-CPMG-NOESY (Fig. 2d) and a (^1^H,^15^N)-HSQC centered on the amino region (Fig. 2e).

In modified *E. coli* tRNA^Asp^, the methyl protons of T54 exhibit a resonance at around 1 ppm that connects with the imino protons of Ψ55, T54 G53 and G52. With this starting point, the remaining resonances of the imino protons in the T-arm were assigned. Two main tertiary interactions are formed via T-loop residues. First, U54 or T54 form a reverse-Hoogsteen base pair with A58. This correlates well with the observed cross peak between the H3 imino proton of T54 and the N7 nitrogen of A58 in the (^1^H,^15^N)-BEST-TROSY-HNN-COSY experiment (Fig. 2c). Additionally, U55/Ψ55 and G18 are implicated in a tertiary interaction connecting the T- and D-loops. H3 iminos of U55 or Ψ55 connect to the imino of G18, allowing the assignment of this upfield shifted imino at 9.46 ppm. Lastly, the H1 imino of Ψ55 resonates with a characteristic ^15^N frequency at 135.2 ppm.

In order to assign the acceptor stem, we took as a starting point the imino of G7 that strongly connects with iminos of the U65-G49 base pair at the extremity of the T-stem. This led us to the sequential assignment of the remaining imino groups in the acceptor-stem (Fig. 2b). G7, G6, and G68 iminos exhibit resonances at very close chemical shifts (∆δ_G7−G6_: ^1^H = 0.11 ppm; ∆δ_G6−G68_: ^1^H = 0.16 ppm), which rendered the assignment delicate. We could only observe G1 resonance peaks in modified samples, which might reflect the dynamic nature of the 3’-ends of tRNAs. Unmodified tRNA^Asp^ indeed exhibits a certain level of structural flexibility in the 3’-end region (Chan et al. 2020).

Lastly, the imino resonance assignment of the anticodon arm was performed using two starting points (Fig. 2b). First the remaining U imino resonance at around 13.3 ppm was assigned to U29 and connects to the iminos of G30 and G42. Second, in the unmodified in vitro tRNA sample, the imino of U25 connects to the imino of G43. Since this particular anticodon arm exhibit strong symmetry in term of base pair sequence (CG-CG-UA-GC-CG), the observation of the connection between U25 and G43 was essential to identify the correct assignment orientation and to allow the assignment of G30, G42 and G39.

Finally, we would like to note that in the modified sample of tRNA^Asp^, Ψ65 is only introduced on approximately half of the tRNA population. This leads to the observation of signals corresponding to the unmodified U65 in the modified sample (Fig. 2b). For example, we can still observe the peak corresponding to unmodified U65 as well as doublets of G7, G49, G51, G52 and G64 due to their spatial proximity to U65.

Chemical shifts for the modified and unmodified *E. coli* tRNA^Asp(GUC)^ have been deposited in the BioMagResBank (http://www.bmrb.wisc.edu) under accession numbers 52476 and 52477, respectively.

### Assignment of *E. coli* tRNA^Val^

The chemical shift assignment of modified *E. coli* tRNA^Val^ has already been reported in previous studies (Hare et al. 1985; Choi and Redfield 1992; Ying et al. 2007; Grishaev et al. 2008; Farjon et al. 2009). To our knowledge, the assignment of the unmodified version of *E. coli* tRNA^Val^ has not been reported, and none of the modified tRNA^Val^ assignments have been deposited to the Biological Magnetic Resonance Bank (BMRB).

In order to assign the imino resonances stemming from the D-arm (Fig. 3b), we took s^4^U8 as a starting point. s^4^U8 displays characteristic downfield shifted imino resonances at frequencies that were assigned at 14.8 ppm for H1 and at 182.8 ppm for N1. Based on intra-base pair NOEs, s^4^U8 connects to iminos of G15, m^7^G46, G22 and U12. This allowed us to assign the remaining G24 and G10 iminos in the D-arm. The imino proton of G10 connects to the imino of a guanine at 10.31 ppm, the latter also connecting to the methyl protons of m^7^G46 at 3.58 ppm. As stated above, a guanine imino signal at similar frequencies was assigned to G45 in tRNA^Ile^ (Jesus et al. 2022) and in tRNA^Asp^ (this study). Without any additional information, we chose to assign this peak to G45 in tRNA^Val^ as well. Although dihydrouridines are not implicated in base-pairing interactions and therefore not protected from exchange with the solvent, the reduced solvent exchange rates of dihydrouridines allow, in this particular case, the detection of a signal corresponding to D17. An imino signal with similar frequencies was also assigned to D20 in *E. coli* tRNA^fMet^ (Biedenbänder et al. 2022).

Starting from the NOESY cross peak between the iminos of U55/Ψ55 and G18, we followed an imino-imino proximity assignment pathway, which led us to the assignment of all imino resonances of modified and unmodified Gs and Us in the T-arm (Fig. 3b). We have also assigned the ^15^N resonance of the H1 imino of Ψ55 at 135.4 ppm, as well as the resonance of the methyl protons of T54 at 0.91 ppm.

In addition, the imino of G49 at the extremity of the T-arm connects with the iminos of U7 and U67 of the acceptor stem. This established a reference point that led to the assignment of all the detectable iminos, from U7 to G1 in the acceptor stem (Fig. 3b).

Lastly, the imino resonance assignment of the anticodon arm was more complicated. U29 was assigned easily as the last remaining unassigned uridine. The imino of U29 connects to iminos of two guanines that resonate at 12.93 ppm and at 12.14 ppm. However, this anticodon arm exhibits perfect symmetry in term of base pair sequence on both sides of U29 (CG-CG-UA-CG-CG), which makes it impossible to assign G42 and G40 in the correct orientation without additional information. Since the anticodon arm is not involved in any complex three-dimensional structural motifs, we relied on a previous report that predicts chemical shift of NH groups in base pair triplet motifs to assign G42 at 12.93 ppm and G40 at 12.14 ppm (Wang et al. 2021). G42 then connects to the imino of G43, and G40 connects to the imino of G39 (Fig. 3b).

Our assignments agree perfectly with previously reported assignments of modified *E. coli* tRNA^Val^ (Hare et al. 1985; Choi and Redfield 1992; Ying et al. 2007; Grishaev et al. 2008; Farjon et al. 2009). Chemical shifts for the modified and unmodified *E. coli* tRNA^Val(UAC)^ have been deposited in the BioMagResBank (http://www.bmrb.wisc.edu) under accession numbers 52480 and 52481, respectively.

### Assignment of *E. coli* tRNA^Phe^

For the assignment of tRNA^Phe^, we relied on the crystal structure of unmodified *E. coli* tRNA^Phe^ (Byrne et al. 2010) to derive the base-pair interactions of both the modified and unmodified tRNA^Phe^ (Fig. 4a).

In order to assign the imino resonances of protected guanines and uridines in the D-arm, we chose, as starting points, U8 and s^4^U8 that exhibit the most downfield shifted imino resonances. The s^4^U8 imino was not detected in the BEST-TROSY experiments performed at 950 MHz. Nevertheless, we observed a weak signal for s^4^U8 in an HSQC experiment performed at 700 MHz, which allowed us to assign its resonances at 14.95 ppm in the ^1^H dimension and 182.2 ppm in the ^15^N dimension. Analyzing the 2D NOESY performed on the modified sample, we observe that s^4^U8 connects with iminos of G15 and G22, with G22 connecting to iminos of m^7^G46 and U12. This allowed the subsequent assignment of the remaining observable imino resonances stemming from the D-arm (Fig. 4b). However, G15 and G46 exhibit no observable iminos in the unmodified sample.

The imino resonance assignment of the T-arm of *E. coli* tRNA^Phe^, from U55/Ψ55 to G65, was performed as described above for tRNA^Val^. Methyl protons of T54 were assigned at 1.035 ppm and connect with imino protons of Ψ55, G53, G52 and G18. The H1 proton of Ψ55 was also assigned at 10.28 ppm for its ^1^H frequency and at 135 ppm for its ^15^N frequency.

In addition, the imino of G65 at the extremity of the T-arm, connects weakly with the imino of G6 from the acceptor stem. G6 was considered as a starting point to assign all the guanines of the acceptor stem (Fig. 4b). However, U66 was not assigned since no imino protons were observable, most likely due to its position at the end of the acceptor helix. In the BEST-TROSY experiment performed on the unmodified sample, the imino proton of G1 resonates at a very close frequency of two other guanines G28 and G29, which makes the peaks in this area not very well defined and required measurements at high field (950 MHz – Table 1).

Finally, we encountered two major difficulties while assigning the anticodon arm. First, it remains three peaks corresponding to guanines on the spectra while four guanines of the anticodon arm are left unassigned. Second, the anticodon arm is composed of a consecutive sequence of GCs and U39/Ψ39 is not visible on the spectra making it challenging to distinguish the right orientation of assignment of the G27, G28, G29 and G30 series in the anticodon arm. Relying on a past report that performed the assignment of the ^1^H NMR spectrum of *E. coli* tRNA^Phe^ (Hyde and Reid 1985) and on the previously mentioned study that predicts chemical shifts of base pair triplet motifs (Wang et al. 2021), we could deduce that G28 and G29 correspond to two superimposed resonances and could subsequently assign all the guanines in the anticodon arm.

Chemical shifts for the modified and unmodified *E. coli* tRNA^Phe(GAA)^ have been deposited in the BioMagResBank (http://www.bmrb.wisc.edu) under accession numbers 52478 and 52479, respectively.

## Data Availability

Chemical shifts data is available at the Biological Magnetic Resonance Bank (http://www.bmrb.wisc.edu).
